# DNA methylation and repressive histones in the promoters of PD-1, CTLA-4, TIM-3, LAG-3, TIGIT, PD-L1, and galectin-9 genes in human colorectal cancer

**DOI:** 10.1186/s13148-018-0539-3

**Published:** 2018-08-06

**Authors:** Varun Sasidharan Nair, Salman M. Toor, Rowaida Z. Taha, Hibah Shaath, Eyad Elkord

**Affiliations:** 1 Cancer Research Center, Qatar Biomedical Research Institute, College of Science and Engineering, Hamad Bin Khalifa University, Qatar Foundation, P.O. Box 5825, Doha, Qatar; 2 Institute of Cancer Sciences, University of Manchester, Manchester, UK

**Keywords:** Colorectal cancer, Immune checkpoints, PD-L1, Galectin-9, DNA methylation, Histone trimethylation

## Abstract

**Background:**

Colorectal cancer (CRC) is the third most commonly diagnosed human malignancy worldwide. Upregulation of inhibitory immune checkpoints by tumor-infiltrating immune cells (TIICs) or their ligands by tumor cells leads to tumor evasion from host immunosurveillance. Changes in DNA methylation pattern and enrichment of methylated histone marks in the promoter regions could be major contributors to the upregulation of immune checkpoints (ICs) in the tumor microenvironment (TME).

**Methods:**

Relative expressions of various immune checkpoints and ligands in colon normal tissues (NT) and colorectal tumor tissues (TT) were assessed by qRT-PCR. The epigenetic modifications behind this upregulation were determined by investigating the CpG methylation status of their promoter regions using bisulfite sequencing. Distributions of histone 3 lysine 9 trimethylation (H3K9me3) and histone 3 lysine 27 trimethylation (H3K27me3) in promoter regions of these genes were assessed by chromatin immunoprecipitation (ChIP) assay.

**Results:**

We found that the expression levels of PD-1, CTLA-4, TIM-3, TIGIT, PD-L1, and galectin-9 were significantly higher in colorectal tumor tissues, compared with colon normal tissues. To study the role of DNA methylation, we checked the promoter CpG methylation of ICs and ligands and found that only CTLA-4 and TIGIT, among other genes, were significantly hypomethylated in TT compared with NT. Next, we checked the abundance of repressive histones (H3K9me3 and H3K27me3) in the promoter regions of ICs/ligands. We found that bindings of H3K9me3 in PD-1 and TIGIT promoters and H3K27me3 in CTLA-4 promotor were significantly lower in TT compared with NT. Additionally, bindings of both H3K9me3 and H3K27me3 in the TIM-3 promoter were significantly lower in TT compared with NT.

**Conclusion:**

This study shows that both DNA hypomethylation and H3K9me3 and H3K27me3 repressive histones are involved in upregulation of CTLA-4 and TIGIT genes. However, repressive histones, but not DNA hypomethylation, are involved in upregulation of PD-1 and TIM-3 genes in CRC tumor tissue. These epigenetic modifications could be utilized as diagnostic biomarkers for CRC.

**Electronic supplementary material:**

The online version of this article (10.1186/s13148-018-0539-3) contains supplementary material, which is available to authorized users.

## Background

Colorectal cancer (CRC) is the third most common cancer worldwide [1]. Approximately 20% of CRC patients show distinct metastases at diagnosis, and the death rate is estimated to be 26% in both genders [1, 2]. The relationship between immune cells and cancer cells within the tumor microenvironment (TME) attains a great interest among researchers. Immune cell-mediated tumor evasion is one of the key mechanisms for the progression and survival of malignant cells [3]. T cells are the chief cytotoxic effector cells that recognize and eliminate tumor cells. Immune response against tumor is initiated by recognition of tumor-antigenic peptides by T cell receptors (TCR) along with co-stimulatory signals, which are required for an effective and prolonged immune response against tumor antigens for successful elimination of malignant cell. In addition to co-stimulatory signals, co-inhibitory signals (immune checkpoints; ICs) are indispensable for maintaining peripheral tolerance and in preventing autoimmunity. The balance between co-stimulatory and co-inhibitory signals determines the amplitude of T cell response [4, 5]. The expression of these ICs is utilized by tumor cells to escape from host immunosurveillance [6, 7].

It has been reported that epigenetic regulation is one of the key mechanisms behind ICs expression in the TME [8]. Three important epigenetic modifications are reported in the colorectal TME; DNA methylation, post-translational modifications in chromatin-protein interactions, and expression of non-coding RNAs [9, 10]. In particular, hypermethylation of the CpG islands (CGIs) enriched in the promoter regions of tumor suppressor genes, induce silencing of these genes [11]. Active demethylation of DNA occurs by the oxidation of 5-methyl cytosine (5-mc) to 5-hydroxymethyl cytosine (5-hmc) and finally to 5-cytosine (5-c) by enzymes belonging to the ten-eleven translocation (TET) family [12]. Mammalian TET family consists of three members; TET1, TET2, and TET3 [12]. It has been reported that promoter demethylation and distribution of repressive histones work together for the upregulation of many genes in cancers [13]. A report showed that the enrichment of repressive histones, histone 3 lysine 9 trimethylation (H3K9me3) and histone 3 lysine 27 trimethylation (H3K27me3) in the promoter regions along with CpG hypermethylation, were the common epigenetic modifications in the colorectal TME [14]. The epigenetic modifications of ICs in colorectal tumor are still not elucidated.

In this study, we investigated expression levels of different immune checkpoints/their ligands, and the epigenetic modifications that could be involved in their upregulation in the colorectal TME. PD-1, CTLA-4, TIM-3, LAG-3, TIGIT immune checkpoints and PD-L1, and galactin-9 ligands were selected due to their important role in tumor immune evasion and their potential as therapeutic targets for immune-mediated therapies. Interestingly, we found that ICs including PD-1, CTLA-4, TIM-3 and TIGIT, and IC ligands including PD-L1 and galactin-9 were significantly upregulated in colorectal tumor tissues (TT), compared with colon normal tissues (NT). Additionally, we found that both DNA hypomethylation and repressive histone binding in the promoter regions are involved in the transcriptional upregulation of CTLA-4 and TIGIT. However, distribution of repressive histones, but not DNA hypomethylation, seems to be involved in the upregulation of PD-1 and TIM-3 in colorectal tumor tissue.

## Results

### Multiple immune checkpoints/ligands are upregulated in colorectal tumor tissue

**Fig. 1:**
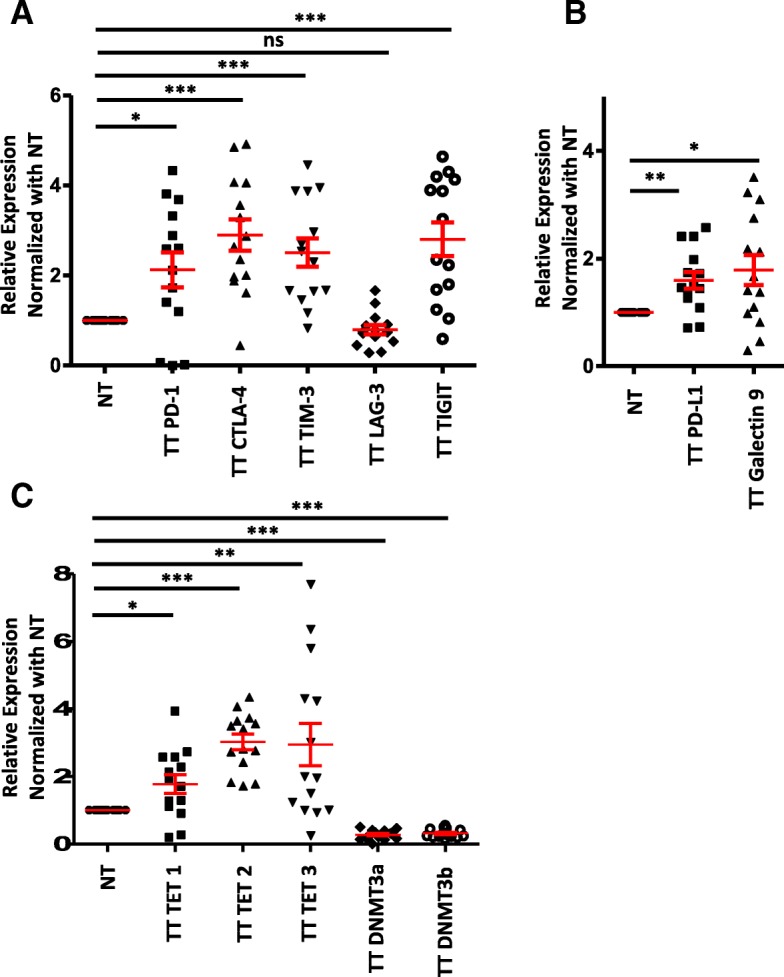
Expression of immune checkpoints/ligands and methylation/demethylation genes in colorectal tumor and normal colon tissues. RNA isolated from tissues from 14 patients was reverse transcribed to cDNA. Quantitative RT-PCR was performed to assess the expression level of immune checkpoints PD-1, CTLA-4, TIM-3, LAG-3, and TIGIT (**a**); immune checkpoint ligands PD-L1 and galectin-9 (**b**); demethylation/methylation enzymes TET1, TET2, TET3, DNMT3a, and DNMT3b (**c**) from both NT and TT. The relative expression of each gene was normalized to β-actin

Reports showed that tumors attain various mechanisms to circumvent host immunosurveillance [15, 16]. One such mechanism is the upregulation of ICs by TIICs and their ligands by tumor cells in the TME. To investigate the transcriptional expression of ICs/ligands in the colorectal TME, we performed real time PCR to determine mRNA levels of ICs/ligands in NT and TT. We found that ICs including PD-1, CTLA-4, TIM-3 and TIGIT, (Fig. 1a) and IC ligands including PD-L1 and galectin-9 (Fig. 1b) were significantly upregulated in TT compared with NT. However, there was no significant change in LAG-3 expression in TT compared to NT (Fig. 1a). These data show that in the colorectal TME, multiple ICs and ligands are upregulated, which may assist tumor cells to evade host immunosurveillance.

### DNA demethylation enzymes are overexpressed in colorectal tumor microenvironment

DNA methylation has a predominant role in the silencing of tumor suppressor genes in the TME, and any imbalance in DNA methylation/demethylation genes could result in disease onset and progression [17]. It has been reported that TET1, TET2, and TET3 exhibit both overlapping and discrete functions [18]. In CRC, somatic mutations have been reported in all three TET proteins [19]. These reports prompted us to check the expression of TET1, TET2, and TET3 and methylation enzymes including DNMT3a and DNMT3b in NT and TT. Interestingly, we found all three TETs were significantly increased and DNMTs were significantly decreased in TT compared with NT (Fig. 1c). Out of all TETs, TET2 was more significantly upregulated in TT compared with NT, indicating that TET2 might play a pivotal role in demethylation than TET1 and TET3 in the colorectal TME (Fig. 1c). The reciprocal expressions of TETs and DNMTs are in line with previous findings that the methylation status of the gene is dynamically regulated by TETs and DNMTs [20, 21].

### Analyses of DNA methylation in the promoter regions of immune checkpoints/ligands in the colorectal tumor microenvironment

**Fig. 2:**
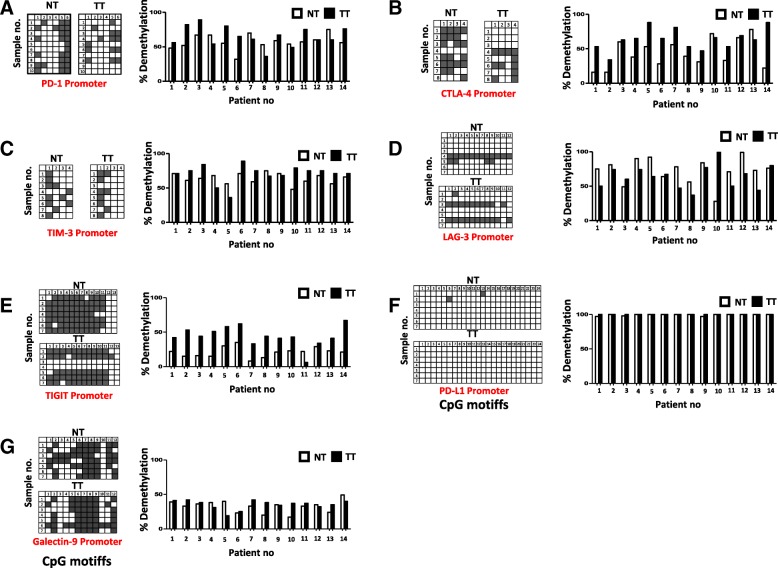
Analyses of CpG methylation of immune checkpoint promoters in colorectal tumor and normal tissues. Representative plots show the CpG methylation of the promoter regions together with bar charts of the demethylation percentages of PD-1 (**a**), CTLA-4 (**b**), TIM-3 (**c**), LAG-3 (**d**), TIGIT (**e**), PD-L1 (**f**), and galectin-9 (**g**) as analyzed by bisulfite sequencing of the genomic DNA isolated from colorectal tumor and normal colon tissues from 14 patients. Methylation status of individual CpG motifs is shown by white (demethylation) or gray (methylation) colors

**Fig. 3:**
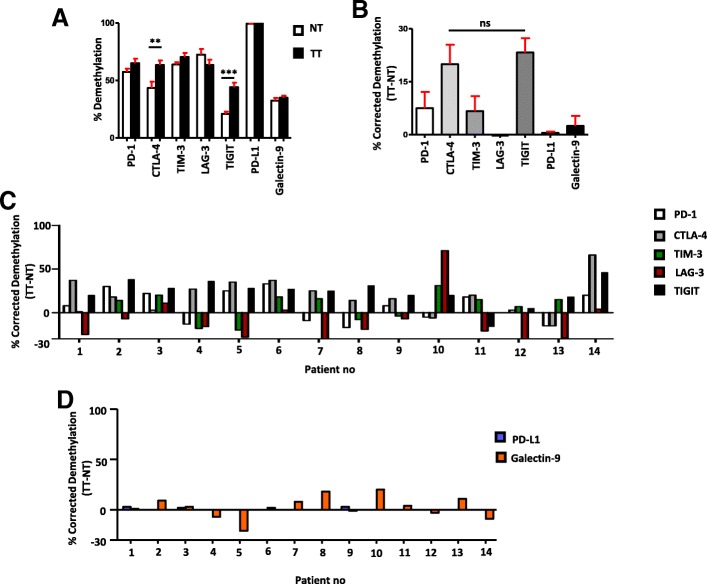
Corrected demethylation percentage of immune checkpoint promoters in tumor tissues. CpG methylation status of the promoter regions of PD-1, CTLA-4, TIM-3, LAG-3, PD-L1, TIGIT, and galectin-9 was analyzed by bisulfite sequencing of the genomic DNA isolated from colorectal tumor and normal colon tissues from 14 patients. A bar diagram shows the average demethylation percentage from the 14 NT and TT samples of each gene (**a**). A bar diagram shows the corrected demethylation percentage of immune checkpoints by subtracting average demethylation percentage of NT from TT (**b**). A bar diagram shows the corrected demethylation percentage of immune checkpoints (**c**) and their ligands (**d**) in 14 individual patients

Hypermethylation of CpG islands (CpGIs) located in the promoter regions have a major role in gene inactivation in the TME and has been defined in almost all malignancies [22]. In order to check the promoter methylation profile of ICs/ligands, we selected CpGIs in the promotors of PD-1, CTLA-4, TIM-3, LAG-3, TIGIT, and PD-L1 as described previously [23]. In addition to this, we also selected 12 CpGIs in the promoter region of galectin-9. We found that the average demethylation percentages of CTLA-4 and TIGIT in TT were significantly higher compared with NT (Figs. 2b, e and 3a). Additionally, the average demethylation percentages of PD-1 and TIM-3 were higher in TT compared to NT, but not significant (Figs. 2a, c and 3a). In contrast, the demethylation of LAG-3 was reduced in TT compared to NT (Figs. 2d and 3a). These results are in accordance with real-time data that LAG-3 was the only gene, which has lower expression in TT compared to NT (Fig. 1a). Additionally, there were no differences in the demethylation percentages for IC ligands, PD-L1 and galectin-9, in TT compared to NT (Figs. 2f, g and 3a). Interestingly, PD-L1 was completely demethylated in both NT and TT (Fig. 2f). This is similar to our findings in breast tumor tissues [23]. These data show that all genes do not follow similar mechanisms for their transcriptional upregulation in the TME. The transcriptional upregulation of CTLA-4 and TIGIT might be under the control of DNA hypomethylation. We also checked the corrected demethylation percentage by subtracting the demethylation percentage of NT from corresponding TT and found that the percentages of CTLA-4 and TIGIT were higher than other genes, and there were no significant differences between them (Fig. 3b). In addition, we checked the corrected demethylation percentages of all genes in individual patients and found that the percentages of CTLA-4 and TIGIT were higher in most of patients compared with other genes (Fig. 3 c, d). These data show that demethylation in promotors might play an important role in the expression of ICs in the TME.

### Analyses of the abundance of repressive histones in the promoter regions of immune checkpoints/ligands in the colorectal tumor microenvironment

**Fig. 4:**
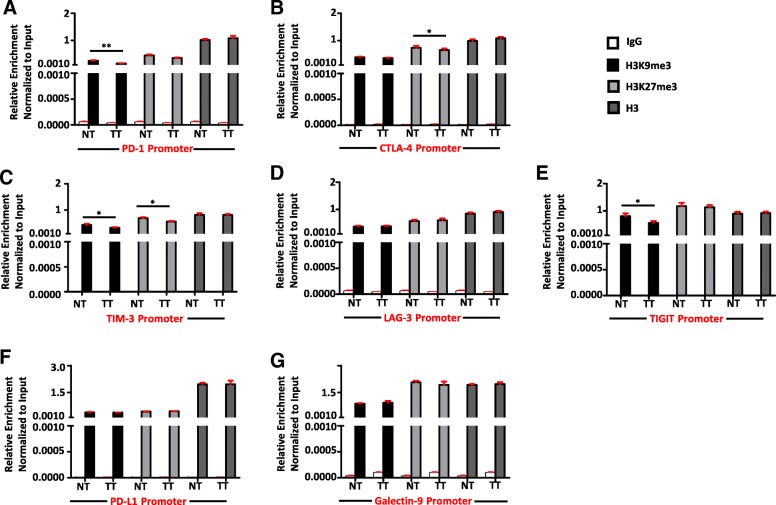
Analyses of distribution of H3K9me3 and H3K27me3 in the promoters of immune checkpoints/ligands in colorectal tumor and normal colon tissues. Cells from five individual NT and TT samples were isolated by enzyme disaggregation. Chromatin was precipitated using anti-H3 as control, anti-H3K9me3, anti-H3K27me3 antibodies, and IgG as negative control. Subsequent qPCR was performed using promoter primers for PD-1, CTLA-4, TIM-3, LAG-3, TIGIT, PD-L1, and galectin-9. Data were normalized to input. ChIP analysis of distribution of H3, H3K9me3, and H3K27me3 at PD-1 (**a**), CTLA-4 (**b**), TIM-3 (**c**), LAG-3 (**d**), TIGIT (**e**), PD-L1 (**f**), and galectin-9 (**g**) promoters are shown

Our DNA methylation data show that the transcriptional upregulation of ICs/ligands are not completely dependent on the hypomethylation of promoter regions. These results prompted us to check the presence of repressive H3K9me3 and H3K27me3 in the promoter regions of PD-1, CTLA-4, TIM-3, LAG-3, TIGIT, PD-L1, and galectin-9 in the colorectal TME by chromatin immunoprecipitation assays. As controls, we precipitated chromatin from both NT and TT with anti-H3 antibody and confirmed that there is no difference in the distribution of H3 in the promoter regions of all ICs/ligands between NT and TT (Fig. 4). We also used rabbit-IgG as an isotype negative control to confirm that there were no non-specific enrichments (Fig. 4). Interestingly, the abundance of H3K9me3 was significantly lower in TT compared with NT in the promoter regions of PD-1 (Fig. 4a) and TIGIT (Fig. 4e), while H3K27me3 was lower in CTLA-4 promotor (Fig. 4b). Moreover, both H3K9me3 and H3K27me3 were significantly lower in TT in TIM-3 promoter (Fig. 4c). Of note, there was no difference in the distribution of either H3K9me3 or H3K27m3e in the promoter regions of LAG-3, PD-L1, and galectin-9 (Fig. 4d, f, g). These data show that in the colorectal TME, abundance of repressive histones in TT was significantly lower in the promoter regions of PD-1, CTLA-4, TIM-3, and TIGIT, which may in turn lead to their transcriptional upregulation in TT compared with NT.

## Discussion

Evidence shows that immune system actively participates in tumor development by promoting the uncontrolled growth of tumor cells [24]. Cancer cells bind to co-inhibitory molecules on T cell surface such as CTLA-4, PD-1, TIM-3, and LAG-3 which in turn secrete immune-suppressive mediators such as IDO (indoleamine 2,3-dioxygenase) to create an immune subversive environment in the TME [25, 26]. We have recently reported that in the breast TME, ICs including PD-1, CTLA-4, TIM-3, and LAG-3 were transcriptionally upregulated in TT compared with NT and both DNA and histone modifications in the TME might be actively involved in this upregulation [23]. Additionally, it has been reported by us and other groups that ICs show elevated expression in the colorectal tumor tissues compared with colon normal tissues [5, 27, 28]. However, the epigenetic modifications behind this upregulation are still not disclosed.

In this study, we found that expression of ICs including PD-1, CTLA-4, TIM-3, TIGIT, and IC ligands including PD-L1 and galectin-9 was significantly higher in colorectal tumor tissues compared with normal tissues (Fig. 1a, b). These findings are in line with our pervious report that the expression of multiple ICs was elevated in the breast TME [23]. In contrast to CRC TME, we did not find IC ligands, PD-L1 and galectin-9 upregulation in the breast TME [23]. These data show that the expressions of ICs/ligands are different in each cancer type, and precise characterization of the ICs and ligands in each cancer type could have prognostic significance. Moreover, we have previously shown that there were more T cell infiltrates in the colorectal TT compared with NT [28]. In this study, we used tissue samples from the same patients that we had used in our previous study [28].

In order to check DNA epigenetic modifications behind the upregulation of ICs/ligands, we checked the expression of demethylation enzymes (TETs) and methylation enzymes (DNMTs) in the tumor and normal tissues and found that the expressions of demethylation enzymes were significantly higher and methylation enzymes were lower in TT (Fig. 1c). It has been reported that the TET protein level was upregulated in solid tumors [29]. These data prompted us to check the CpG methylation profile of the promoter regions of ICs/ligands. We found that the promoter regions of CTLA-4 and TIGIT were significantly hypomethylated in TT compared with NT. These data suggest that not all ICs are following similar epigenetic modifications to upregulate their expression in the TME. Additionally, there was no significant difference in the demethylation percentage in LAG-3 promoter between NT and TT (Fig. 3a). These results are similar to our previous findings in the breast TME that the promoter regions of PD-1, CTLA-4, and TIM-3 were significantly hypomethylated in TT compared with NT and no change in LAG-3 [23]. Compared to our previous study [23], we found that in both NT and TT of colorectal and breast tumors, the CpGs in the promoter region of PD-L1 have been totally demethylated (Fig. 2f), but the relative expression of PD-L1 was significantly higher only in the colorectal TT compared with NT (Fig. 1b). Taken together, our data suggest that the transcriptional upregulation of ICs/ligands does not solely depend on promoter CpG hypomethylation but also on malignant type.

In addition to CpG methylation, we also investigated whether the histone modifications also participate in the upregulation of ICs/ligands in the colorectal TME. It has been reported that promoter region hypermethylation is often associated with H3K9me3 and H3K27me3 for transcriptional silencing [30]. Herein, we checked H3K9me3 and H3K27me3 markings in the promoter regions of PD-1, CTLA-4, TIM-3, LAG-3, TIGIT, PD-L1, and galectin-9 (Fig. 4). In accordance with our previous findings in breast tumors [23], the distribution of H3K9me3 was lower in colorectal TT of PD-1 (Fig. 4a) and H3K27me3 was lower in TT of the CTLA-4 (Fig. 4b) and TIM-3 (Fig. 4c) promoter regions compared with NT. Moreover, there was no change in distribution of either H3K9me3 or H3K27me3 in the promoter regions of LAG-3, PD-L1 and galectin-9 in colorectal TT compared to NT (Fig. 4d, f, g). We have reported that in the breast TME, the relative expression of LAG-3 was higher in TT compared with NT, and also the distribution of both H3K9me3 and H3K27me3 was lower in TT compared with NT [23]. Of note, in CRC tumor tissue, there was no upregulation in the expression of LAG-3 and also no difference in the distribution of either H3K9me3 or H3K27me3 in TT compared to NT (Figs. 1a and 4d). Taken together, these data show that the expression of ICs and the epigenetic modifications in the TME differ in different malignancy types.

## Conclusions

This study advances our knowledge in both molecular and epigenetic modifications behind the upregulation of ICs/ligands in the colorectal TME. We showed that multiple ICs/ligands including PD-1, CTLA-4, TIM-3, TIGIT, PD-L1, and galectin-9 are upregulated in the colorectal TME. The epigenetic modifications, including DNA hypomethylation and less abundance of H3K9me3/H3K27me3 in the promoter regions, could be responsible for their upregulation. Moreover, the transcriptional upregulation of CTLA-4 in tumor tissue might be under the control of both DNA hypomethylation and lower H3K27me3 enrichment, while DNA hypomethylation and lower H3K9me3 enrichment regulate TIGIT expression. Additionally, lower enrichment of H3K9me3 or both H3K9me3 and H3K27me3 markings could be behind the upregulation of PD-1 and TIM-3 expressions in the CRC TME, respectively. These examinations of promoter DNA methylation and distribution of repressive histones in different ICs/ligands could be further utilized as a diagnostic tool for colorectal cancer.

## Methods

### Sample collection

Tumor tissues (TT) and adjacent non-cancerous normal tissues (NT) were obtained from 14 colorectal cancer patients who underwent surgery. All patients provided written informed consent prior to sample collection and none of the patients included in this study received any treatment prior to surgery. Table 1 shows the clinical and pathological characteristics of all patients. The study was executed under ethical approval by the Qatar Biomedical Research Institute, Doha, Qatar (Protocol no. 2017–006). All experiments were performed in accordance with relevant guidelines and regulations.

**Table 1:** Characteristic features of study population

S no.	Patient ID	Age	Sex	Histological grade	TNM stage
1	CRC 09	56	F	Poorly differentiated	I
2	CRC 12	39	F	Moderately differentiated	IIA
3	CRC 14	41	F	Poorly differentiated	IIIC
4	CRC 15	46	M	Moderately differentiated	IIC
5	CRC 16	67	M	Moderately differentiated	I
6	CRC 18	52	M	Moderately differentiated	IIIB
7	CRC 21	62	M	Poorly differentiated	IIIC
8	CRC 22	41	F	Poorly differentiated	IIIB
9	CRC 26	60	M	Moderately differentiated	IIA
10	CRC 28	39	F	Poorly differentiated	IVB
11	CRC 29	41	F	Moderately differentiated	IIA
12	CRC 30	40	M	Well differentiated	IIIB
13	CRC 32	39	F	Moderately differentiated	IIIB
14	CRC 33	36	M	Moderately differentiated	IIIC

### RNA and DNA isolation

RNA and DNA were isolated using RNA/DNA/Protein Purification Plus Kit (Norgen Biotek Corp, Ontario, Canada) as per manufacturer’s instructions, from 14 fresh-frozen TT and their corresponding NT. Briefly, frozen tissues were grinded thoroughly using mortar and pestle with adequate amount of liquid nitrogen. Tissue fragments were then resuspended with lysis buffer and incubated at 55 °C for 10 min. DNA extraction was then performed using the DNA extraction column. The flow-through from DNA extraction was used for RNA purification using RNA extraction column. The flow-through from RNA extraction was then used for protein extraction using the same column. RNA and DNA concentrations were measured using Nanodrop 2000c (Thermo Scientific, MA, USA), and aliquots were stored at − 80 °C.

### Quantitative real-time PCR (RT-qPCR)

One microgram of RNA from each sample was reverse transcribed into cDNA using QuantiTect Reverse Transcription Kit (Qiagen, Hilden, Germany). RT-qPCR was performed on QuantStudio 7 Flex qPCR (Applied Biosystems, CA, USA) using PowerUP SYBER Green Master Mix, and all data were normalized to β-actin. Non-specific amplifications were checked by using melting curve and agarose gel electrophoresis. The relative changes in target gene expression were determined using comparative threshold cycle (CT) method 2^-ΔΔCT^ between NT and TT. The primers were designed using Primer3 (http://www.ncbi.nlm.nih.gov/tools/primer-blast/) and Harvard Primer Bank (http://pga.mgh.harvard.edu/primerbank/). Primer sequences are provided in Additional file 1: Table S1a.

### CpG methylation analysis by bisulfite sequencing

CpG methylation analyses were performed through bisulfite sequencing as previously described [23]. Briefly, genomic DNA was extracted from NT and TT, and bisulfite treatment was performed using the EZ DNA Methylation Kit (Zymo Research, Irvine, CA, USA). PCR was then performed on the bisulfite-treated DNA for amplification of the promoter regions of PD-1, CTLA-4, TIM-3, LAG-3, TIGIT, PD-L1, and galectin-9 using hot start TaKaRa Taq DNA polymerase (TaKaRa Bio, Shiga, Japan). PCR primers were designed using MethPrimer software (http://www.urogene.org/methprimer/index1.html). Primer details are provided in Additional file 1: Table S1b. PCR products were cloned into the pGemT-easy vector (Promega, Madison, USA) using DNA Ligation Kit, Mighty Mix (TaKaRa Bio). Ten individual clones from each sample were purified using Wizard® Plus SV Minipreps DNA Purification System (Promega) and sequenced using M13-reverse/forward primers (Additional file 1: Table S1c). The promoter regions amplified for CpG methylation profile in this study were as previously described [23].

### Enzyme disaggregation of tumor and normal tissues for cell isolation

Cell suspensions for ChIP experiments were obtained from frozen NT and TT of five CRC patients by enzyme disaggregation (ED), as previously described [23]. Briefly, thawed tissues were first washed with phosphate-buffered saline (PBS) and mechanically cut into small fragments (2–4 mm) using a surgical scalpel. Tissues were then suspended into RPMI-1640 with 1% penicillin/streptomycin and enzyme cocktail consisting of 1 mg/ml collagenase and 100 μg/ml hyluronidase type V (all from Sigma-Aldrich, UK) and incubated at 37 °C under slow rotation for 60 min. The resulting cell suspension was then passed through a 100 μm BD Falcon cell strainer (BD Biosciences, Oxford, UK), washed with serum free RPMI-1640, and resuspended in RPMI-1640 enriched with 10% FCS and 1% penicillin/streptomycin for further analyses.

### Chromatin immunoprecipitation assay (ChIP)

ChIP analysis was performed using Magna ChIP A/G chromatin immunoprecipitation kit (Merck Millipore, MA, USA) as per manufacturer’s protocol on cells isolated from NT and TT by ED. Briefly, nuclear lysate was prepared as per manufacturer’s protocol and sonicated using Covaris S2 system (Covaris, MA, USA) to make small DNA fragments (100–200 base pairs) and then incubated with ChIP grade anti-Histone H3 rabbit mAb (Active Motif, CA, USA), anti-Histone H3 (tri methyl K9) rabbit mAb (Abcam Cambridge, UK), and anti-Histone H3 (tri methyl K27) rabbit mAb (Abcam). Isotype-matched control antibodies were used as negative controls. Immune complexes containing DNA fragments were precipitated using Magna A/G beads (supplied with the kit). Relative enrichment of target regions in the precipitated DNA fragments was analyzed by qPCR using PowerUP SYBER Green Master Mix (Applied Biosystems) on QuantStudio 7 Flex platform (Applied Biosystems). Sequences of primers are listed in Additional file 1: Table S1d. All data were normalized to input controls. Non-specific amplification was checked by using melting curve and agarose gel electrophoresis.

### Sanger sequencing

Purified plasmid DNA samples were subjected to sequencing using 3130X Genetic Analyzer (Applied Biosystems). Cycle sequencing reactions of samples were performed using M-13 forward/reverse primers and BigDye Treminator V3.1 (Applied Biosystems), using thermal conditions: 95 °C for 5 min, 35 cycles of 95 °C for 30 s, and 60 °C for 4 min. DNA was precipitated after PCR reaction using 125 mm EDTA and 95% ethanol and incubated at − 20 °C for 30 min. DNA was then washed twice with 70% ethanol followed by denaturation using formaldehyde. Denatured DNA was then loaded into analyzer for sequencing. Sequencing data were analyzed using Bisulfite Sequencing DNA Methylation Analysis (BISMA) software (Jacobs University, Germany).

### Statistical analyses

Statistical analyses were performed using GraphPad Prism 5 software (GraphPad Software, USA). Paired *t* test was carried out on samples within groups that passed the Shapiro–Wilk normality test. Nonparametric/Wilcoxon matched-pairs signed-rank tests were performed on samples that did not pass normality test. A *P* value of < 0.05 was considered statistically significant. The *P* values are represented as ***P < 0.001, **P < 0.01, *P < 0.05. The data are presented as mean + standard error of the mean (SEM).

## Additional file

Additional file 1: **Table S1.** Primer sequences used in this study. (DOCX 21 kb)

## Abbreviations

ChIP: Chromatin immunoprecipitation
CpGI: CpG islands
CTLA-4: Cytotoxic T lymphocyte-associated protein 4
DNMT: DNA methyltransferase
DNMTi: DNA methyltransferase inhibitor
ED: Enzyme disaggregation
H3K27me3: Histone 27 lysine 9 trimethylation
H3K9me3: Histone 3 lysine 9 trimethylation
LAG-3: Lymphocyte-activation gene 3
NT: Normal tissue
PBS: Phosphate-buffered saline
PD-1: Programmed cell death protein-1
PD-L1: Programmed death-ligand 1
RT-qPCR: Quantitative real-time PCR
SEM: Standard error of the mean
TET: Ten-eleven translocation dioxygenase
TIGIT: T cell immunoreceptor with Ig and ITIM domains
TIM-3: T cell immunoglobulin and mucin-domain containing-3
TT: Tumor tissue
